# Increased Acetylcholinesterase Expression in Bumble Bees During Neonicotinoid-Coated Corn Sowing

**DOI:** 10.1038/srep12636

**Published:** 2015-07-30

**Authors:** Olivier Samson-Robert, Geneviève Labrie, Pierre-Luc Mercier, Madeleine Chagnon, Nicolas Derome, Valérie Fournier

**Affiliations:** 1 Centre de recherche en innovation sur les végétaux, Université Laval, Québec, G1V 0A6, Canada; 2 CÉROM, Centre de recherche sur les grains Inc., Saint-Mathieu-de-Beloeil, Québec, J3G 0E2, Canada; 3 Institut de Biologie Intégrative et des Systèmes (IBIS), Université Laval, Québec, QC G1V 0A6, Canada; 4 Département des Sciences Biologiques, Université du Québec à Montréal, Montréal, Québec, H3C 3P8, Canada

## Abstract

While honey bee exposure to systemic insecticides has received much attention, impacts on wild pollinators have not been as widely studied. Neonicotinoids have been shown to increase acetylcholinesterase (AChE) activity in honey bees at sublethal doses. High AChE levels may therefore act as a biomarker of exposure to neonicotinoids. This two-year study focused on establishing whether bumble bees living and foraging in agricultural areas using neonicotinoid crop protection show early biochemical signs of intoxication. Bumble bee colonies (*Bombus impatiens*) were placed in two different agricultural cropping areas: 1) control (≥3 km from fields planted with neonicotinoid-treated seeds) or 2) exposed (within 500 m of fields planted with neonicotinoid-treated seeds), and maintained for the duration of corn sowing. As determined by Real Time qPCR, AChE mRNA expression was initially significantly higher in bumble bees from exposed sites, then decreased throughout the planting season to reach a similar endpoint to that of bumble bees from control sites. These findings suggest that exposure to neonicotinoid seed coating particles during the planting season can alter bumble bee neuronal activity. To our knowledge, this is the first study to report *in situ* that bumble bees living in agricultural areas exhibit signs of neonicotinoid intoxication.

In the last ten years, honey bee (*Apis mellifera*) colonies worldwide have suffered major losses^1^,^2^. Climate change, beekeeping practices, genetic weakening, loss of nutritional diversity due to monocultures, parasites, pathogens, pesticides and other factors, acting alone or in combination, are suspected to play a role in ongoing honey bee losses^2^,^3^. The current extensive use of pesticides is one of the most frequently debated, and appears to be a major contributor to honey bee colony losses^4^,^5^. Nicotine-related insecticides, or neonicotinoids, have notably been a growing cause for concern, and many studies have found evidence of their adverse effects on bees^6^,^7^,^8^,^9^,^10^.

Introduced in the mid-1990s, neonicotinoids have become the most widely used class of insecticides on the planet and represent more than 30% of the world’s insecticide market share^11^. These neurotoxic insecticides have systemic properties that allow the active ingredients to be taken up by a plant’s root system and translocated to all of its parts, including inflorescences^11^. Due to their water-soluble properties, their use as a soil treatment and seed coating has become wide-spread. By 2008, neonicotinoids accounted for 80% of the global insecticidal seed treatment market share^11^. In the United States, more than 140 million acres of land was sown with neonicotinoid-treated seeds in the year 2010 alone^12^. Since the widespread adoption of neonicotinoids in agricultural environments, frequent bee kill events in spring have been reported^6^,^13^,^14^. Recent studies revealed that planting neonicotinoid-coated corn releases high levels of clothianidin and thiamethoxam into the environment^6^,^13^,^14^,^15^,^16^,^17^,^18^,^19^. Dandelions visited by foraging bees and growing in proximity to coated corn fields have also been found to be contaminated by neonicotinoids^6^. Hence, emission of contaminated dust during corn sowing appears to be one of the main routes of exposure to neonicotinoids during spring.

Neonicotinoids and some of their metabolites are potent agonists and act selectively on post-synaptic nicotinic acetylcholine receptors (nAChRs) of insects’ central nervous systems^20^,^21^. Mimicking the natural neurotransmitter, they bind with very high affinity to these receptors and trigger a neuronal hyper-excitation, which can, under a lethal dose, cause an insect to die within minutes^21^. Under sublethal doses, bees have been shown to begin succumbing 15 days after the initial exposure^22^. nAChRs are an assemblage of subunit combinations, and subtypes of nAChRs in insects show very different pharmacological profiles compared to nicotinic receptors in vertebrates^20^. As a result, neonicotinoids have shown a particular selective toxicity for insects over mammals and fish^20^,^21^. Before the advent of neonicotinoids, organophosphates and carbamates shared more than 80% of the insecticidal market^11^. However, these older insecticide classes act directly on acetylcholinesterase (AChE). They are equally toxic to mammals and insects because they exert an inhibitory effect on the regulatory enzyme AChE, which is responsible for hydrolysis of the neurotransmitter acetylcholine (ACh)^23^. Neonicotinoids, however, act on nAChRs and, by mimicking ACh, stimulate the insect’s metabolism to keep producing AChE as a natural response to end the neural transmission in synapses. Increased AChE activity has been reported in response to exposure to neonicotinoids, both in honey bees and other arthropods^24^,^25^. To our knowledge, with the rare exception of one pyrethroid (deltamethrin)^26^, neonicotinoid compounds are the only agrochemicals that cause an increase in AChE activity. Consequently, AChE represents a useful biomarker to reveal honey bee exposure to this class of insecticides in their environment.

Given the ecological and economic importance of pollinators, they have received extensive attention from the scientific community, policy makers, the media and the general public. Unfortunately, attention has focused largely on honey bees, and research has only recently broadened to encompass native pollinators as well^4^,^7^,^27^,^28^,^29^,^30^,^31^,^32^,^33^. Yet from an ecological standpoint, native bees are of greater concern than honey bees since they are usually more efficient pollinators of native plants with which they have coevolved^34^. The majority of native bee species are solitary and thus do not accrue numerous advantages from the sheer size of a colony. Moreover, honey bee colonies benefit from management by beekeepers. For example, intoxicated or unproductive queens can be replaced, pollen supplements can be supplied and parasite populations can be controlled to ensure the colony survives and thrives. Since eusocial or semisocial native bees are unmanaged by man, their colonies cannot be nursed back to health by beekeepers. Wild bee populations therefore seem more vulnerable to direct anthropogenic pressures (fragmentation of habitats, loss of nutritional diversity and the use of pesticides) than managed honey bee colonies. An alarming number of studies have reported the steady decline of wild bees in North America as well as in Europe^35^,^36^. Furthermore, although research on neonicotinoids has been abundant, many agencies and NGOs have stressed the need to improve knowledge on the impacts of these insecticides, in particular on native bees and under natural conditions, about which little is known^17^,^18^,^19^.

The objective of this study was therefore to investigate whether native pollinators living in agricultural areas show signs of intoxication during spring, as recent studies demonstrated that exposure to neonicotinoid compounds is particularly high at this time of the year^6^,^13^,^14^,^15^,^16^,^17^,^18^,^19^. Traditionally, intoxication is determined by chemical analyses of bees that are already dead. However, bodies of dead native bees are almost impossible to find in the wild, and the potential number of specimens would not be large enough to be able to collect the quantity of biological material required for chemical analysis. As minute quantities of insect growth regulators, below the detection limits of analytical chemistry, can adversely affect bee performance and trigger disorders in colony dynamics^37^, the same can be expected from the highly toxic neonicotinoids. Furthermore, most neonicotinoids are rapidly metabolized within the insect’s body, making the parent compounds very difficult to identify in dead bees collected from the field. Thus, the use of a biomarker would be particularly useful in detecting exposure to neonicotinoids. As such, AChE expression was used in this study as a probable biomarker to rely on specimens alive at the time of their capture. This approach also allows intoxications to be detected before they reach lethal levels, and provides the opportunity to take counteracting measures. Managed bumble bees were selected to represent native pollinators of the same species for the purpose of establishing whether pollinators other than honey bees show biochemical signs of intoxication at sublethal levels.

## Results

**Figure 1:**
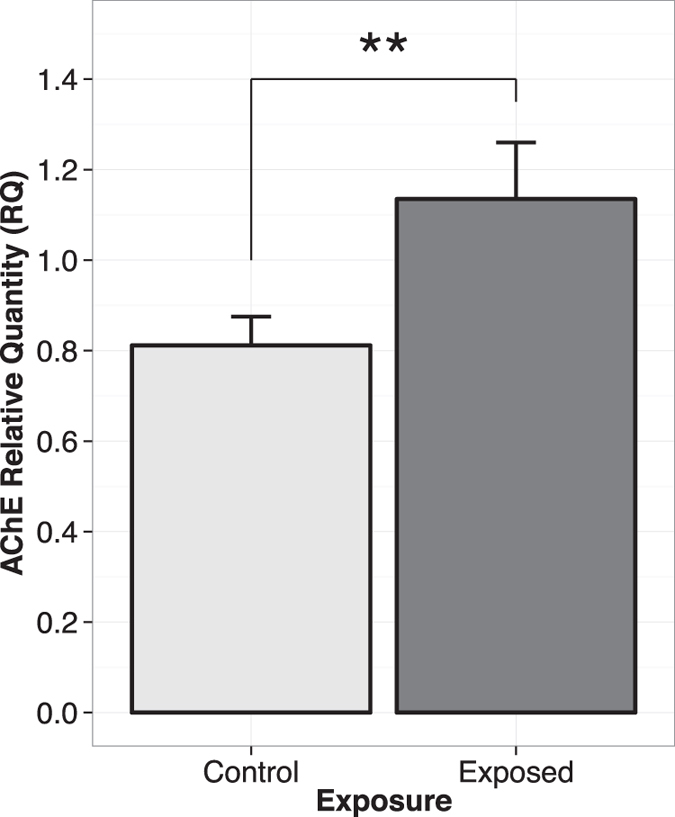
AChE relative quantity in bumble bee heads as obtained from RealTime qPCR analyses (n = 32 for control and n = 52 for exposed) for both years combined. Control treatment corresponds to bumble bees whose colony is at least three kilometers distant from a field planted with neonicotinoid-treated seeds. Exposed treatment corresponds to bumble bees from a colony located within 500 meters of a field planted with neonicotinoid-treated seeds.

In 2012, 290 bumble bees were dissected, for a total of 29 samples (10 bumble bee brains per sample), of which 9 originated from control and 20 from exposed colonies. In 2013, a total of 550 bumble bees underwent dissection, resulting in 55 samples, 23 from control and 32 from exposed colonies. The relative quantity of AChE mRNA in brain extracts for 2012 and 2013 was significantly higher in bumble bees from colonies located in agricultural areas where neonicotinoid-treated seed use was ubiquitous (*F*_*1,21*_ *=* *9.27; p* *=* *0.006*) (Fig. 1). Mean AChE mRNA relative quantity in the control group (agricultural area without neonicotinoid seed treatments) was 0.811 ± 0.065, and 1.135 ± 0.127 in exposed groups. This represents an increase of 40% in comparison to the baseline AChE expression level established by the control groups. There was no significant difference in AChE relative quantity between study sites or between years.

**Figure 2:**
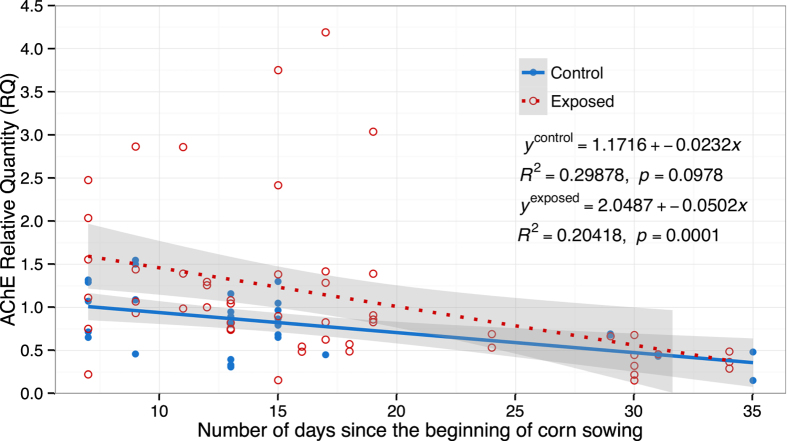
AChE relative quantity in bumble bee heads as a function of days since the beginning of corn planting. Open circles and dotted line refer to control bumble bees’ AChE levels whereas filled circles and continuous line are for exposed bumble bee samples. Day 0 corresponds to the first day of corn planting and bumble bee colonies were placed in the field at this date. First samples were collected at Day 7 to ensure bumble bees had gotten acquainted with their new environment. In 2012, last bumble bee samples were collected at Day 35, and in 2013, at Day 24, when corn planting had been completed.

Interaction between treatment and time had a p value slightly higher than the accepted standard value of 0.05 (*F*_*1,56*_ *=* *3.26; p* *=* *0.0764*), but would still be considered biologically relevant in view of the fact that this research was field-based^38^. A new repeated measures model was designed to define the temporal effect of each treatment individually (Fig. 2). Bumble bee colonies were placed in the field at day 0 (t = 0), the first samples of bumble bees were collected seven days later (t = 7), and the last samples of bumble bees were collected when corn planting was considered completed (t = 35). The intercept for the AChE relative quantity for bumble bees from the exposed group was significantly higher than that of bumble bees from the control group (*F*_*2,21*_ *=* *4.15; p* *=* *0.0303*). The AChE relative quantity for the control group tended to decrease slightly, but not significantly, over time (*F*_*2,57*_ *=* *2.43; p* *=* *0.0978*), while there was a strong decrease over the same period for the exposed group (*F*_*2,57*_ *=* *10.46; p* *=* *0.0001*). No persisting relative differences between AChE expression rates were observed once corn planting had been completed.

## Discussion

Neonicotinoid insecticides are ubiquitous in agricultural areas. Their extensive use has been linked with increased honey bee colony losses, and sublethal doses have been found to adversely affect honey bee behaviour, foraging, homing, olfaction, learning, fecundity and even colony development^4^,^7^,^8^,^29^,^30^,^31^,^33^,^39^,^40^,^41^,^42^,^43^,^44^,^45^. Monitoring AChE expression is a first step toward better understanding the extent to which native pollinators are exposed to neonicotinoids in their natural environment. Our study represents the first report of an increase in AChE for a pollinator species other than the honey bee under entirely natural conditions.

The results presented here provide evidence that bumble bees living and foraging near agricultural fields of neonicotinoid-treated corn are impacted at the level of AChE expression throughout the planting period. Routes of exposure to neonicotinoids for bees are multiple. Although mechanisms were not quantified in this study, contaminated dust emitted during planting constitutes the probable primary source of exposure in spring. Previous studies have found extremely high levels of clothianidin and thiamethoxam in vacuum planter exhaust manifolds that would affect pollinators in the immediate vicinity^6^,^15^,^46^. Corn planting occurs in many fields simultaneously, and may produce sufficient contaminated dust to affect extensive agricultural areas where pollinators live and forage. Direct contact with contaminated soil and vegetation or consumption of contaminated pollen, nectar and water might also contribute to intoxication, as they may have been contaminated by drifting dust or uptake of residual neonicotinoids in the soil by root systems^6^,^16^. Cumulative exposure from these multiple routes may be sufficient to alter AChE production in bumble bees. In the context of a more extensive field study on neonicotinoids, water samples from temporary puddles in the vicinity of our study sites were collected during the planting period for pesticide residue analyses. Previously published results^10^ show that residues of neonicotinoid insecticides were present in 100% of samples taken from neonicotinoid-coated corn fields, whereas these compounds were never found in control areas. Furthermore, water quality surveys conducted in intensive corn production areas confirmed that neonicotinoid insecticides were present in 100% of monitored water puddles^47^ and waterways^48^. These results demonstrate that neonicotinoids are indeed present in the exposed sites and that the bumble bees from our study, constrained by their flight range (within 2 km)^49^,^50^, were well exposed to neonicotinoids. As for deltamethrin, the only other pesticide known to increase AChE^26^, it was never found in either control or exposed areas^10^,^48^. This is understandable, since deltamethrin is rarely used today in corn and soy production in Quebec. Also, when it is used, this product is applied later in the season (July or August), a time when our field study had been completed.

Significant variations in biomarker expression have been found in the honey bee, depending on the developmental stage of queens and workers^51^. Moreover, the age-related division of labour specific to honey bee workers involves enhanced learning and memory capabilities for acquisition of new tasks. Forager bees have lower AChE expression, which is associated to increased cholinergic neurotransmission and overall cerebral activity^52^. In the honey bee, this biological variability in AChE expression can be controlled and reduced by sampling only a specific caste with specific duties, such as foragers^53^. In bumble bees the mechanism underlying the division of labour does not seem to be as discrete as in honey bees^54^, however. Bumble bee polyethism appears to be related to both age and body size. Previous research has shown that older and larger bumble bee workers are generally more likely to forage, whereas younger and smaller ones usually perform “in-nest” tasks^55^,^56^. This distinct division of labour may pose a problem when using biomarkers that are affected by the developmental stage of workers, since sampling a specific caste with specific duties does not guarantee a definite developmental stage. Although bumble bee body size has been shown to affect the expression of the foraging gene implicated in the division of labour^57^, no study has shown that biomarkers, including AChE, are affected by this natural variation in body size. Also, bumble bee foragers tend to be of similar age in young colonies and thus possess similar AChE production^58^. In our study, all colonies were of similar age and were, furthermore, placed in the field as soon as possible after rearing. By the end of the experiment, colonies were no more than two months old and foragers were still thought to be of similar age with consequently similar AChE expression. One study has demonstrated that adult bumble bees have higher AChE levels than both larval and pupal stages, but found that AChE expression in adults is not significantly affected by age and does not differ between foragers and nurses^59^. It appears that sampling a specific caste with specific duties, such as foragers, reduces much of the biological variability in AChE expression for bumble bees just as for honey bees.

Additionally, our results imply a relationship between exposure to neonicotinoid compounds and time elapsed since the beginning of corn planting. Bumble bees from control sites (3 km or more from fields planted with n-treated seeds) initially had a much lower AChE level than bumble bees from exposed sites. Bumble bees were potentially exposed to neonicotinoids for a maximum of seven days, because first samples were collected a full week after colonies had been placed in the fields. As mentioned above, our results suggest that this exposure seems to be sufficient to raise AChE levels in bumble bees from exposed sites. On the other hand, control colonies were located in an environment free of neonicotinoid treated-seeds, which would explain their initially lower AChE expression. AChE expression in bumble bees from control colonies decreased slightly, but not significantly, as the planting season progressed and reached its minimal level at the end of the experiment, when planting was completed. We hypothesize that stressors such as colder weather, the need to become acquainted with their new environment and the initial lack of foraging resources (very few flowers are in bloom during early spring) tend to increase bumble bees’ AChE levels in spring. After this initial surge, AChE drops to a more typical level. In bumble bees from exposed colonies, AChE expression steadily decreased over time as corn planting progressed, reaching a similar endpoint as that of bumble bees from the control colonies when corn planting was completed. These results suggest that as corn planting progresses, less area remains to be sown and thus the amount of contaminated dust released in the environment becomes less and less important. As exposure diminishes, so do bumble bees’ AChE levels. When corn planting is completed and dust emission ceases, AChE expression is comparable to that in bumble bees from control and exposed colonies (Fig. 2).

An earlier field study showed an increase in AChE in honeybee foragers exposed to neonicotinoids in seed treated corn fields during pollen shed compared to foragers from organic corn fields^25^. These field results were confirmed by laboratory assays showing an increase in AChE expression in caged honeybees exposed to known sublethal doses of imidacloprid and clothianidin^25^. Our study demonstrates that a biomarker such as AChE acts in a similar way in pollinators other than honeybees, in this case bumble bees, when exposed to neonicotinoid insecticides.

## Conclusion

Although the impacts of neonicotinoids on honey bees have been thoroughly investigated, remarkably little research has focused on this problem in wild pollinators. To our knowledge, this is the first time that an increase in AChE has been reported for a pollinator species other than the honey bee under field conditions. Although the exact exposure mechanisms implicated are difficult to identify, it appears that cumulative exposure to neonicotinoids during planting operations of coated seeds is sufficient to alter AChE expression in bumble bees. These findings confirm that native pollinators, such as bumble bees, are exposed to neonicotinoids during the corn planting season simply by living and foraging in agricultural areas. Establishing intoxication thresholds while measuring AChE expression levels would be a next logical step in understanding the consequences of this exposure for pollinators.

## Methods

### Experimental setting

Field trials took place in two neighbouring administrative regions in the southern part of the province of Quebec, in eastern Canada. The two regions, Montérégie (45° 37’ 10” N, 72° 57’ 30” W) and Estrie (45° 24’ 00” N, 71° 53’ 03” W), share similar weather conditions and historically have a high level of agricultural land-use. Montérégie is the province’s major corn and soybean producing region, constituting nearly 60% of the area devoted to these crops. Since 2008, nearly 100% of Quebec’s corn seeds are treated with neonicotinoid coating prior to planting, and more than two-thirds of soybean crops are similarly treated^48^. The Estrie region produces comparatively very little corn and soybean, and its agricultural profile is more evenly distributed among different food crops. In the Montérégie region, study sites (n = 9 for 2012, n = 7 for 2013) were located within 500 m from a corn field planted with neonicotinoid-treated seeds (exposed treatment) in a landscape (3 km radius) where such fields are ubiquitous. Study sites selected in the Estrie region (n = 3 for 2012, n = 7 for 2013) were located at least 3 km from any field planted with neonicotinoid-treated seeds (control treatment). Study sites selected were also locations where beekeepers annually place honey bee colonies and that are known honey producing areas. This ensured that bumble bees could forage successfully in proximity to their nest. Moreover, in the context of a more extensive field study on neonicotinoids, chemical analyses for pesticide residues in water puddles (within 1 km of study sites)^10^ and dead honey bees (Samson-Robert *et al*., under review) were carried out using the same experimental sites and during the same sampling periods for both years (2012 and 2013). These analyses allowed us to evaluate and compare the level of exposure to neonicotinoids between control and exposed sites. Results showed that control sites were exempt of neonicotinoid insecticides as these were never detected in samples from water puddles samples (N = 15) or dead honey bees (N = 16). In contrast, for exposed sites, residues of neonicotinoids were detected in 100% of water puddle samples (N = 59) and, furthermore, half the samples of dead honey bees (N = 58) contained residues of clothianidin (range from 0.128 ng/bee to 1.92 ng/bee; mean = 0.727 ± 0.095 ng/bee) and thiamethoxam (range from 0.128 ng/bee to 0.64 ng/bee ; mean = 0.228 ± 0.04 ng/bee). Therefore, it is safe to assume that bumble bee colonies assigned to control sites were not exposed to neonicotinoid compounds whereas those from exposed sites were.

### Bumble bee colonies

Commercial *Bombus impatiens* colonies were purchased from Koppert Biological Systems (Scarborough, Ontario, Canada). Colony strengths were assessed (number of hatched workers) prior to shipping and, since colonies were of similar strength, were randomly and evenly assigned to either treatment (exposed or control). Colonies were maintained in the field for the duration of corn planting, from the beginning of May until mid-June (approx. 5 weeks). A single bumble bee colony was placed at every study site in 2012 (12 colonies), and one quad unit of four bumble bee colonies per site in 2013 (56 colonies). During the first year of the study, when only one bumble bee colony was placed on-site, it was at times impossible to capture the required number of bumble bee foragers, which in turn limited the number of samples available for AChE analyses. After the first year of sampling, it was deemed necessary to increase the number of colonies to ensure that a sufficient number of bumble bee foragers would be available for specimen collection, without putting too much stress on the colony through this reduction of its population. Colonies were left untouched in the field for seven days to ensure that foragers became accustomed to their new environment and to minimize residual stress from hive transportation. Also, since colonies contained between 50 to 70 worker bees at the time they were initially placed on-site, collecting ten forager bees was not feasible without strongly stressing the colonies and impeding their development. Subsequently, when possible, ten foragers were collected from each site every 48 hrs until corn planting operations were completed (4 weeks in 2012 and 3 weeks in 2013). To facilitate collection, the hive entrance was obstructed by lowering the “bee-home” door, and foragers buzzing around the entrance were captured by sweeping nets. Captured foragers were immediately killed on dry ice and kept frozen for transportation to the laboratory. Specimens were maintained at −80 °C until dissection.

### Brain dissection

Since previous histochemical analyses of AChE in honey bees have shown this enzyme to be distributed predominantly in the central nervous system, brain tissue was targeted for analysis in this study^59^. Dissections were performed by removing the face plate (from the epistomal sulcus to the vertex) and lifting the brain away from the head capsule. To ensure minimal RNA degradation, dissection procedures had to be completed within one minute from the moment a specimen was taken out of the freezer. Dissected brains (10 per sample) were placed in 1 mL of RNA isolation reagent (TRI Reagent, Sigma T9424) and were homogenized by vortexing. Brain homogenates were kept at −80 °C until needed for RNA extraction.

### Quantitative real time PCR

RNA isolation was carried out following the method described by Quanta-Biosciences (QScript One-Step SYBR Green RT-qPCR kit), with minor modifications. RNA was extracted by adding 200 μL of chloroform per 1 mL of Tri Reagent solution. RNA was purified with 250 μL of isopropanol and 250 μL hypersaline solution (1.2 M trisodium citrate; 0.8 M NaCl) per 1 mL of Tri Reagent solution. A NanoDrop spectrophotometer was used to determine the purity and concentration of RNA samples. qPCR was performed using the following primers: RPL13a control (GenBank , accession number XM_623810), forward primer, TGGCCATTTACTTGGTCGTT, reverse primer, GAGCACGGAAATGAAATGGT; AChE-2 (accession number FJ666117.1), forward primer, CCTCCATGCGGTACAGAGTT, reserved primer GTCCCTGAGCCATCTGAGAG. Results are expressed in RQ values, the relative quantity of the RNA template in the original samples.

### Statistical analysis

Initial models were constructed for each individual year. As trends in AChE expression and conclusions were similar for 2012 and 2013, both years were pooled for analysis. AChE expression data set was analyzed using a generalized randomized block design (GRBD) with repeated measures. Each experimental unit consisted of a sample of 10 bumble bee foragers. GRBD refers to the randomization of study sites within treatments (exposed and control) and within blocks comprised of both study years (2012 and 2013). In this model, treatments and dates were specified as fixed effects, while years and study sites were considered as random. Bumble bee specimens were collected from the same colonies at repeated intervals throughout the corn planting season. Repeated measures design was used to address the longitudinal nature of the data set. The correlation structure for observations within the same experimental unit was selected based on Akaike Information Criterion (AIC). The normality assumption was tested using the Shapiro-Wilk’s statistic, and the homogeneity of variances was verified using the usual residual plots. Box-Cox power transformation was used to remedy violation of the model’s assumptions. Statistical analyses were performed with the lme() function in the nlme package of R software (V 3.0.2) with the significance level set at 0.05. However, considering the fact that this research was field-based, a p value inferior to 0.1 would have been considered significant^38^.

## Additional Information

**How to cite this article**: Samson-Robert, O. *et al*. Increased Acetylcholinesterase Expression in Bumble Bees During Neonicotinoid-Coated Corn Sowing. *Sci. Rep*. **5**, 12636; doi: 10.1038/srep12636 (2015).
